# Tonic–clonic seizure during the ultrasound-guided stellate ganglion block because of an injection into an unrecognized variant vertebral artery: A case report

**DOI:** 10.1097/MD.0000000000018168

**Published:** 2019-11-27

**Authors:** Fan Lu, Jie Tian, Jifu Dong, Kexian Zhang

**Affiliations:** Department of Anesthesiology, Sichuan Cancer Hospital & Institute, Sichuan Cancer Center, School of Medicine, University of Electronic Science and Technology of China, Chengdu, China.

**Keywords:** seizure, stellate ganglion block, ultrasound-guided

## Abstract

**Rationale:**

Recent years have witnessed a marked improvement in the safety and accuracy of nerve blocks with the help of ultrasound and other visualization technologies. This study reports a challenging case of a severe complication during the ultrasound-guided stellate ganglion block.

**Patient concerns:**

A 28-year-old male patient with refractory migraine complained episodic pulsatile pain with photophobia, haphalgesia of the scalp for 3 years.

**Interventions:**

Ultrasound-guided stellate ganglion block with 4 ml of 1% lidocaine was administrated.

**Outcomes:**

A sudden loss of consciousness and tonic–clonic seizure was occurred after negative aspiration and test dose. Further sonographic examination revealed a variation in the left vertebral artery, which remained unrecognized during the needle insertion because of its sliding ability under the differential pressure applied by the probe.

**Lessons:**

Inadvertent intra-arterial injection of a local anesthetic agent could be minimized under the ultrasound guidance with various protective strategies, including the determination of any prior variation, optimizing the block route, maintaining a constant probe pressure, and using saline for the test dosage. This case resulted in the implementation of new protocols of the ultrasound-guided stellate ganglion block in our department.

## 1 Introduction

A stellate ganglion block is a standard diagnostic and treatment modality for reflex sympathetic dystrophy and Meniere disease. It also demonstrates a good effect on sympathetically maintained headaches, including migraines.^[1–3]^ Compared with a blind procedure, the ultrasound guidance is a reliable method to decrease complications arising from an intravascular injection and nerve injury because of its advantages in the direct visualization of the trachea, blood vessels, thyroid, and bony surfaces.^[4]^ However, a successful ultrasound-guided stellate ganglion block warrants proficiency in anatomy, pre-procedural planning, and inserting technologies to evade any possible injury of the proximity structures.^[5]^ In addition, intravascular injections could cause mild to severe complications, from tinnitus to even cardiac arrest.^[6,7]^ Here, we report the case of a patient who demonstrated symptoms of sudden loss of consciousness and tonic–clonic seizure during the ultrasound-guided stellate ganglion block, despite us taking several preventive measures such as test dose, negative aspiration, and good cooperation of the patient.

## 2 Case presentation

A 28-year-old Asian male patient was admitted to our hospital for a refractory migraine of 3 years. He complained hours of pulsatile pain with photophobia, haphalgesia of the scalp, and fatigue during each attack. His medical history was unremarkable, and his magnetic resonance imaging and computed tomography angiography revealed no imaging anomaly. Accordingly, he was treated with naproxen (0.5 g qd) and flunarizine hydrochloride (10 mg qd) with poor improvement. Thus, treatment with an ultrasound-guided stellate ganglion block on each side a time was planned as a complementary therapy.

**Figure 1:**
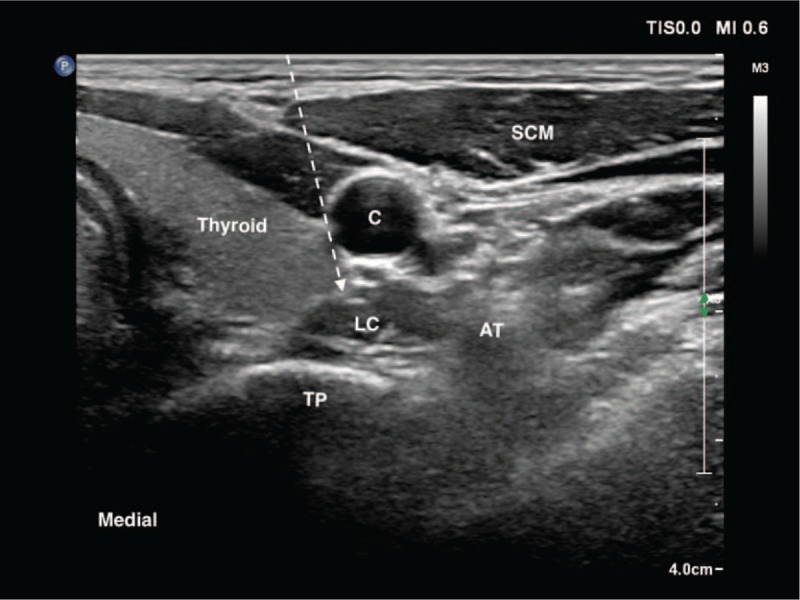
The planned puncture path route at the sixth cervical vertebral level. AT = anterior tubercle, C = carotid artery, LC = longus colli muscle, SCM = sternocleidomastoid muscle, TP = transverse process. Puncture path (dotted arrow).

With the patient's informed consent, the first block was successfully performed on the right side with temporary Horner syndrome, decreasing attacks on the first day. Thus, the next block for the other side was administrated the day after. During the practice, the patient laid in the supine position with a thin pillow under his shoulder to ensure the extension of the anterior neck. Before the procedure, the patient was instructed to raise his hand if he felt any discomfort but was not allowed to speak or swallow. He was monitored by pulse oximetry and noninvasive blood pressure. We adopted the in-plane approach because of the clear ultrasonography at the level of the 6th cervical vertebra (C6) transverse process and prepared 4 ml of 1% lidocaine with a 22-gauge needle and 12-MHz linear probe. During the procedure, the needle was supposed to be inserted between the carotid artery and thyroid toward the prevertebral fascia anterior to the longus colli muscle (Fig. 1). However, we lost track of the tip when the inserting the needle, which passed the fascia of the artery and thyroid. Hence, the operator cautiously jiggled the needle to detect the tip. After the tip was confirmed by the concomitant jitter of the target tissue (the surface of the longus colli muscle), 0.5 ml of 1% lidocaine was injected; however, the injectate could not be clearly visualized, prompting the operator to slowly withdraw the needle and decide using the in-plane approach instead. The patient suddenly raised his hand and lost consciousness followed by approximately 2-minute tonic–clonic seizure. The patient was immediately ventilated manually with 100% oxygen by a facemask, and an intravenous line was set up followed by an injection of 2-mg midazolam. The patient quickly returned to calm and natural breathing state, and, after 10 minutes of somnolence, regained complete consciousness but could not recall the previous experience.

**Figure 2:**
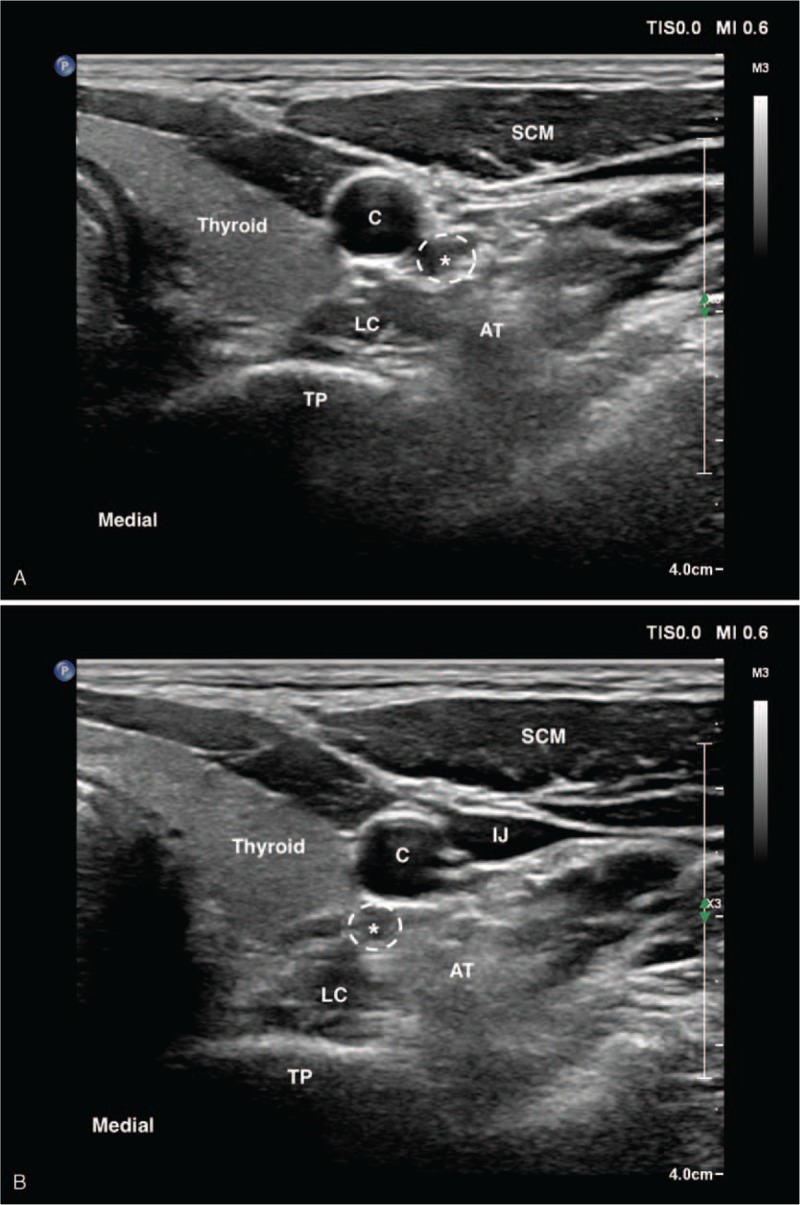
The change in the position of the left vertebral artery under the differential pressure of ultrasound probe. (a) The ultrasound image at the C6 level with higher pressure on the probe; (b) The ultrasound image at the C6 level with less pressure on the probe. AT = anterior tubercle, C = carotid artery, IJ = internal jugular vein, LC = longus colli muscle, SCM = sternocleidomastoid muscle, TP = transverse process. ^∗^Vertebral artery (white dotted circle).

**Figure 3:**
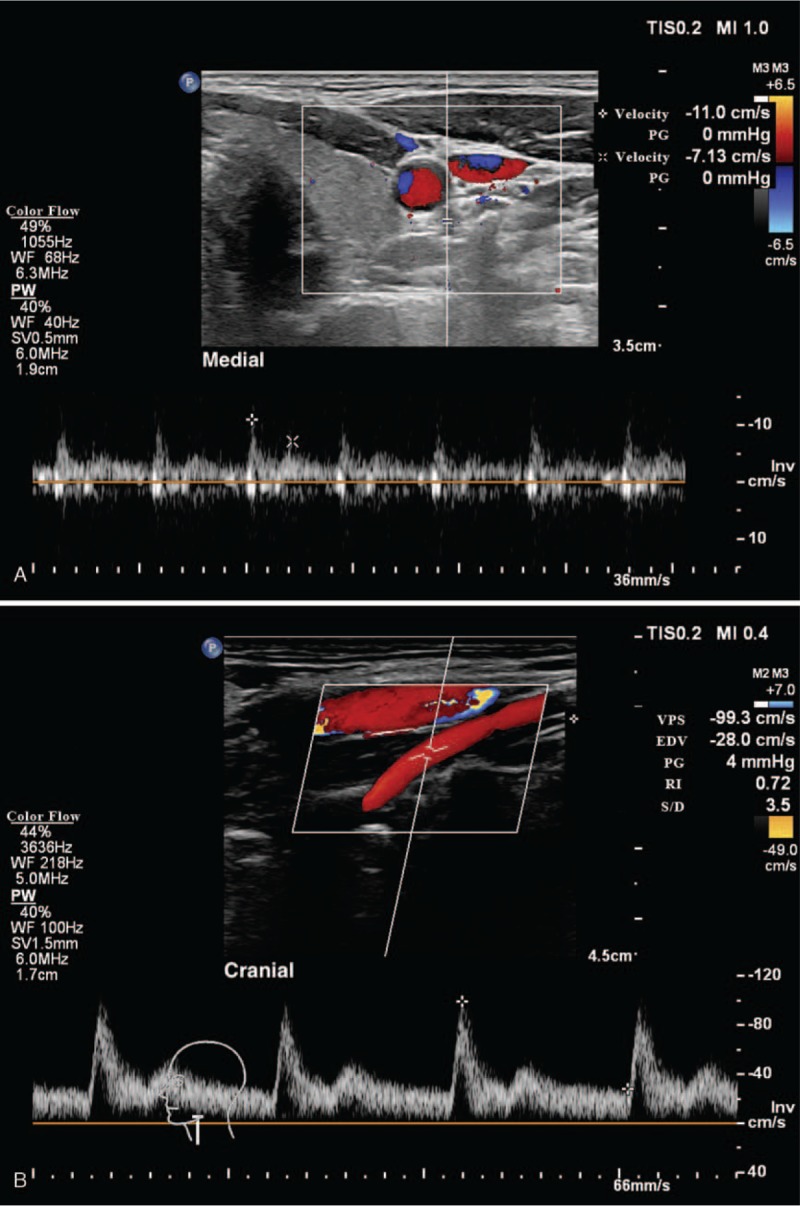
The color Doppler ultrasound image of the vertebral artery. (a) The M-mode ultrasound image of the vertebral artery at C6 level; (b) The color Doppler ultrasound image showed the left vertebral artery entered the transverse foramen at C5.

Later, we used ultrasound to re-examine the patient, but found no hematoma. However, further examination revealed that a 6-mm-diameter artery at the C6 level was in the posteromedial of the carotid artery, which went completely unrecognized at the initial scanning, and could easily slide to the lateral side when the operator pressurizes the probe (Fig. 2). Then, we traced cranially to determine the entry of the artery into the transverse foramen of the 5th cervical vertebra (C5) (Fig. 3), which was preliminarily determined as a variant vertebral artery. The patient was discharged after 2-hour of observation and was followed up for 2 months. No complaints of dizziness, tinnitus, and dysphagia were reported.

## 3 Discussion

This case reports the rare occurrence of a severe complication during the ultrasound-guided stellate ganglion block. Although ultrasound remarkably enhances the safety of nerve blocks, avoidable negligence can be encountered even by senior operators when there is a lack of complete protocols. In our case, an intra-arterial injection was confirmed by intoxicated reactions of a local anesthetic agent and ultrasonography of the unrecognized left vertebral artery.

Intra-arterial injection, intravenous injection, and regional absorption could cause the systemic local anesthetic toxicity. The onset and severity of complications highly depend on the injection routes and dosage of the local anesthetic agent. The lowest dose of lidocaine-associated seizure during intravenous regional anesthesia was 1.4 mg/kg,^[8]^ and only large volumes (5–10 ml) of a local anesthetic agent could cause systemic toxicity within 5 minutes during an inadvertent intravenous injection.^[9,10]^ However, central nervous system symptoms might occur very rapidly after a bolus of an intra-arterial injection. The minimum toxic dose of lidocaine injected into the vertebral artery is approximately 16.8 mg for adults with a body weight of 70 kg.^[11]^ In our case, the tonic–clonic seizure occurred only after the 5-mg lidocaine injection, which was lower than any other reports.^[11,12]^ The rapid injection might be accountable for the toxic central plasma concentration of local anesthetic agents. Mechanism research revealed that the central toxicity of lidocaine originates in areas of thalamocortical neurons and could result from evoked high-threshold Ca^2+^ currents.^[13]^

In this case, intra-arterial injections occurred despite the absence of the blood reflux. Some studies have reported complications followed by negative aspiration,^[11,14,15]^ suggesting that the single aspiration is not entirely reliable to determine the location of the needle tip. The best method is always to ensure good visualization of the entire needle, especially the tip, under the ultrasound guidance, and re-confirm the tip position by the spread of the liquid sonolucent in real-time.

The stellate ganglion block is considered to be safer at the C6 level to avoid the proximity of the vertebral artery, inferior thyroid artery, and pleura. However, the risk of this procedure could be profoundly exacerbated with the absence of the transverse foramen, as in the case of a vertebral artery variation. Approximately 2% to 10% of the vertebral artery entered into the transverse foramen at a level other than C6, with the most common variation entrance at C5.^[16–18]^ In our procedure, the anatomical variation was undetected at the C6 level during the pre-scanning because the high pressure conducted by the probe pressed the vertebral artery to the lateral side of the carotid artery. However, during the out-of-plane puncture, this uncovered vertebral artery slid to the needle path because of pressure variations. In addition, the muscular neck of the patient made it even harder to detect the tip in the out-of-plane approach, eventually resulting in the intra-arterial injection. Thus, maintaining the constant pressure of the ultrasound probe is imperative to retain an optimal insertion path under the ultrasound guidance. Moreover, it is not recommended to inject a local anesthetic agent to verify the location of the unvisualized tip even if it is possibly near the target areas. Instead, normal saline can work as a good alternative injectate to visualize the needle tip with no occurrence of local anesthetic intoxication.^[19]^

**Figure 4:**
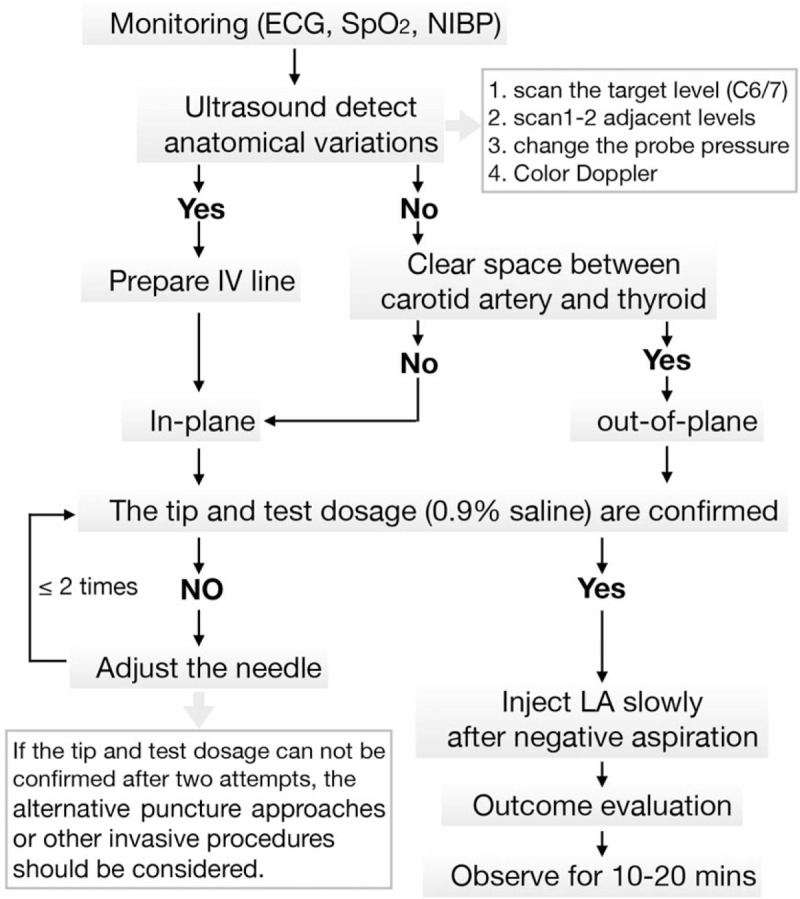
The new protocol for ultrasound-guided stellate ganglion block.

A new protocol for ultrasound-guided stellate ganglion block was implemented in our department (Fig. 4). The out-of-plane technique is primarily applied to the superficial nerve block, especially when the repeated blocks to the same target are required. This approach shortens the puncture path route and time but is only recommended for experienced operators and in the absence of any anatomical variation of the target areas. The vertebral artery is not the only cause for the intra-arterial injection in the neck during the stellate ganglion block, the presence of the ascending cervical artery, deep cervical artery, and inferior thyroid artery is recorded in the proximity of puncture levels.^[20]^ Thus, the in-plane technique or semi-in-plane technique is essential for patients with high risks of puncture-related complications. Meticulous pre-scanning plays a vital role in the block strategy decision, and it is necessary to move the probe cranially and caudally from the target level, assisted by tilting and pressure variation to eliminate the possibility of variations. For patients with known variations, it is preferable to comprehensively assess the benefits of this invasive procedure in the first place, educate patients to prove good cooperation, put an intravenous line beforehand, and ensure visualizing the whole needle during the block. If the tip of needle and the test injectate were not confirmed after 2 attempts, the alternative procedures should be considered.

## Acknowledgments

The authors acknowledge the patient presented case report, and thank Dr. He Huang for editorial assistance for this manuscript.

## Author contributions

**Conceptualization:** Kexian Zhang.

**Methodology:** Kexian Zhang.

**Supervision:** Kexian Zhang.

**Validation:** Jifu Dong.

**Writing – original draft:** Jie Tian, Fan Lu.

**Writing – review & editing:** Jie Tian, Fan Lu.
